# Novel Enantiopure Sigma Receptor Modulators: Quick (Semi-)Preparative Chiral Resolution via HPLC and Absolute Configuration Assignment

**DOI:** 10.3390/molecules21091210

**Published:** 2016-09-10

**Authors:** Marta Rui, Annamaria Marra, Vittorio Pace, Markus Juza, Daniela Rossi, Simona Collina

**Affiliations:** 1 Department of Drug Sciences, Medicinal Chemistry and Pharmaceutical Technology Section, University of Pavia, Viale Taramelli 12, Pavia 27100, Italy; marta.rui01@universitadipavia.it (M.R.); annamaria.marra@unipv.it (A.M.); daniela.rossi@unipv.it (D.R.); 2 Department of Pharmaceutical Chemistry, University of Vienna, Althanstrasse 14, Vienna 1090, Austria; vittorio.pace@univie.ac.at; 3 Corden Pharma Switzerland LLC, Eichenweg 1, Liestal 4410, Switzerland; markus.juza@cordenpharma.com

**Keywords:** sigma receptor (SR) modulators, amylose- and cellulose-derived CSPs, chiral resolution, enantioselective HPLC, electronic circular dichroism (ECD)

## Abstract

The identification of novel pan-sigma receptor (SR) modulators, potentially useful in cancer treatment, represents a new goal of our research. Here, we report on the preparation of novel chiral compounds characterized by a 3-C alkyl chain bridging an aromatic portion to a 4-benzyl-piperidine moiety. All of the studied compounds have been prepared both in racemic and enantiomerically-pure form, with the final aim to address the role of chirality in the SR interaction. To isolate and characterize enantiomeric compounds, high-performance liquid chromatography (HPLC) procedures were set up. A systematic analytical screening, involving several combinations of chiral stationary and mobile phases, allowed us to optimize the analytical resolution and to set up the (semi-)preparative chromatographic conditions. Applying the optimized procedure, the enantiomeric resolution of the studied compounds was successfully achieved, obtaining all of the compounds with an enantiomeric excess higher than 95%. Lastly, the absolute configuration has been empirically assigned to enantiopure compounds, combining the electronic circular dichroism (ECD) technique to the elution order study.

## 1. Introduction

**Figure 1:**
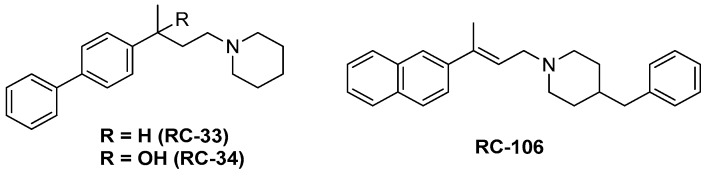
Structures of RC-33, RC-34 and RC-106.

Sigma receptors (SRs) are involved in different pharmacological and pathological pathways. They modulate cell survival and excitability and sub-serve many critical functions in the human body. To date, two subtypes have been identified: Sigma 1 (S1R) and Sigma 2 receptors (S2R). S1Rs are overexpressed in the central nervous system (CNS), while S2Rs are overexpressed in tumor cells and tissues in proliferation [1]. Accordingly, S1R modulators could be useful for the treatment of several CNS diseases, such as anxiety, depression, schizophrenia, drug addiction, Parkinson’s and Alzheimer’s diseases [2], whereas S2R ligands could have a relevant role in cancer diagnosis and therapy [3]. Since some research groups identified an overexpression of S1R in different typologies of cancer cells and demonstrated a strict correlation between S1R and this pathological manifestation, S1R has been also proposed as a potential target for treating cancer conditions [4,5,6]. As a result, therapies that have S1R and S2R as targets might play a role against a wide spectrum of cancer types. Indeed, recent literature highlights the anticancer potential of ligands able to bind both receptor subtypes, called pan-SR ligands [7]. The work presented here falls into this field, and it is a part of our ongoing research focused on the identification of novel SR modulators. Along the years, we prepared and studied a wide compound library of SR modulators [8]. The SAR analysis evidenced that a good affinity towards both SRs is related to the presence of a bulky aminic moiety [9,10]. Particularly, the 4-benzylpiperidine derivative RC-106 (Figure 1) is characterized by a good affinity toward both S1R and S2R (Ki S1R = 12.0 nM ± 6.0; Ki S2R = 22.0 nM ± 1.1). Moreover, the SAR studies of chiral SR modulators showed that small changes in the ligand structure markedly influence the ability of SRs to discriminate the enantiomeric form. For example, racemic and enantiomeric RC-33 (Figure 1) interact in a non-stereoselective manner with SRs, both enantiomers showing a similar affinity toward both S1R and S2R [11,12,13]. A different behavior was observed for the analogue RC-34, characterized by the presence of an electronegative element (the only structural difference with respect to RC-33). In this case, the (*S*)-configured enantiomer resulted in being the eutomer, with the following eudismic ratio (ER, K_i__distomer_/K_i__eutomer_):ER_S1R_ = 8.3 and ER_S2R_ = 2.5 [14]. According to these results, the capability of SRs to discriminate between the enantiomers of a ligand seems to be strictly related to its structural features [15].

In this paper, we report on the preparation of the potential chiral SR modulators **1**–**6**, characterized by an alkyl or alcoholic spacer bridging an aromatic moiety to the 4-benzyl-piperidine portion (Table 1) and discuss in detail our efforts to develop easy-to-use chiral chromatographic methods, this approach being effective for both analytical and preparative purposes [16,17,18,19]. Moreover, we report on absolute configuration assignment studies of enantiomerically-pure **1**–**6**, combining electronic circular dichroism (ECD) and elution order studies. Our final aim is to have enantiopure **1**–**6** in a sufficient amount for the biological investigation, in order to study the intriguing aspect of the influence of chirality in SR interaction.

## 2. Results and Discussion

### 2.1. Synthesis

**Scheme 1:**
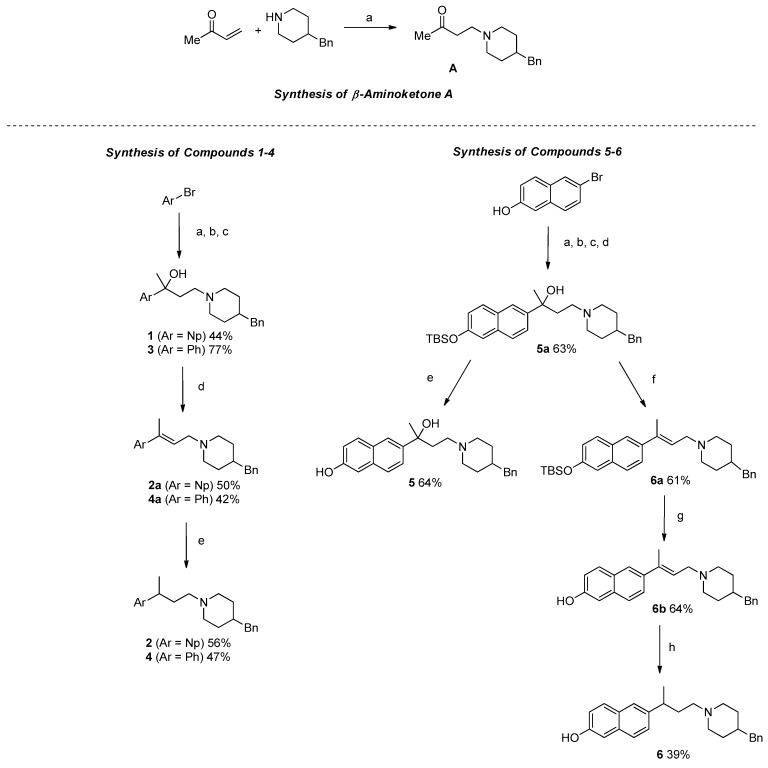
Synthesis of Compounds **1**–**6**. Experimental conditions: β-aminoketone A: (**a**) glacial acetic acid, abs EtOH, rt; Compounds **1**–**4**: (**a**) *t*-BuLi, anhydrous Et_2_O, −78 °C to rt; (**b**) ketone **A**, −78 °C to 0 °C; (**c**) H_2_O rt; (**d**) Cu(OTf)_2_, (CF_3_O)_2_O, anhydrous DCM, 0 °C to rt; (**e**) H_2_, Pd(0)/C 10% (*p*/*p*), abs EtOH, rt; Compounds **5**–**6**: (**a**) TBDMSCl, DMF; (**b**) *n*-BuLi, THF, −78 °C; (**c**) ketone **A**, −50 °C; (**d**) NH_4_Cl (aq.), rt; (**e**) TBAF, CH_2_Cl_2_, 0 °C; (**f**) TFAA, Cu(OTf)_2_ (2 mol·%) CH_2_Cl_2_, 0 °C to rt; (**g**) TBAF, CH_2_Cl_2_; (**h**) H_2_ (1 atm) Pd/C (10 mol %) EtOH, rt.

As reported in Scheme 1, the synthesis of 4-(4-benzyl-piperidin-1-yl)-butan-2-one, common intermediate (**A**), was accomplished in a good yield via the Michael addition of 4-benzyl piperidine to but-3-en-2-one in absolute ethanol and glacial acetic acid. Briefly, Compounds **1** and **3** were obtained adopting a nucleophilic addition strategy of in situ-generated organolithium species to ketones. The appropriate aryl-bromine was lithiated with an excess of *tert*-butyllithium (*t*-BuLi) in anhydrous diethyl ether (Et_2_O) at −78 °C, followed by the addition of **A**: the desired tertiary alcohols **1** and **3** were obtained in practical yields. Subsequently, the so-obtained alcohols were subjected to dehydration with trifluoroacetic anhydride under copper(I) triflate catalytic conditions [20]. Purification on alumina column chromatography provided the olefins **2a** and **4a**, which were hydrogenated (Pd (0)/C 10% (*p*/*p*)) to give the corresponding reduced amines **2** and **4**. The same tactic was applied for accessing naphthol derivatives **5** and **6**. However, prior to lithiation, the protection of the aromatic alcohol as TBS-ether was required to prevent interference during the metalation step. Additional points merit mention: (1) keeping the temperature at −50 °C after the quenching with **A**, (2) employing THF as the solvent and (3) using the less basic n-BuLi as the lithiating agent resulted in being beneficial to maximize the yield. Lastly, under identical conditions to those ones reported for the synthesis of **2** and **4**, Compound **6** was obtained in a good yield.

### 2.2. Analytical Screening and Development of a Scalable Resolution Method

The strategy we adopted for obtaining enantiopure **1**–**6** is based on our knowledge and extensive experience in chiral liquid chromatography that led us to have a wide range of chiral analytical and (semi-)preparative columns in-house. As a first choice, we used amylose- and cellulose-based CSPs immobilized on silica gel chiral columns, due to their wide solvent compatibility, versatility and robustness. In details, Chiralpak IC (cellulose tris(3,5-dichlorophenylcarbamate immobilized on silica gel) and Chiralpak IA (amylose tris(3,5-dimethylphenylcarbamate, immobilized on silica gel) have been used, eluting with alcohols (MeOH or/and EtOH) or with *n*-hep in the presence of polar modifiers (EtOH or IPA), DEA 0.1% and TFA 0.3%.

Therefore, the HPLC screening protocol of Table 2 was applied, and only when the results of this screening were encouraging, but not fully successful, the mobile phase was slightly modified in order to achieve a compound baseline separation. Since no satisfactory chiral separation for all compounds was obtained, conventional cellulose-based chiral column Chiralcel OJ-H (cellulose tris(4-methylbenzoate, coated on silica gel) was also experimented with, again eluting with alcohols (MeOH or/and EtOH) or *n*-hep in the presence of IPA as polar modifier and adding DEA 0.1%. Results of the analytical screening are reported in Table 3, Table 4 and Table 5 and are expressed as retention (k), selectivity (α) and resolution (Rs) factors.

Chiralpak IC provided a baseline separation of Compounds **1**, **2**, **4** and **6**, even if the chromatographic conditions were not suitable for a productive scale-up (Table 3). In detail, no enantioresolution was observed eluting with alcohols (data not shown), while modest or good values of α and Rs were obtained eluting with *n*-hep/EtOH or *n*-hep/IPA, even if the retention times (t_R_) are quite long (t_R_ of the second eluted enantiomer ranging from 30–90 min). Moreover, to solve Compound **1**, a slight modification of the mobile phase composition of the screening protocol, eluting with *n*-hep/IPA (92/8, *v*/*v*), has been necessary. Nevertheless, a modest resolution was achieved (α = 1.12 and Rs = 1.44).

Chiralpak IA gave rise to better results, being effective in resolving Compounds **1**–**6** (Table 4). Interestingly, the presence of *n*-hep in the mobile phase was essential for the separation of **1**, **3** and **5** and gave rise to quite long retention times (t_R_ of the second eluted enantiomer ranging from 15–65 min). Conversely, **2**, **4** and **6** (endowed with an alkyl spacer) have been successfully resolved eluting with pure MeOH in short times (t_R_ second eluted enantiomer less than 10 min).

On the basis of these results and keeping in mind that our purpose is to set up an economic and productive preparative enantiomer separation for **1**–**6**, we turned our attention to a further analytical screening, using the Chiralcel OJ-H column. Baseline separation of Compounds **1**–**3** and **5** has been obtained in short retention times, less than 12 min (referred to the second eluted enantiomer). Results are reported in Table 5. Interestingly, high enantioselectivity and good resolution have been achieved eluting only with alcohols, while no separation occurs eluting with *n*-hep, with the only exception of Compound **1**.

**Figure 2:**
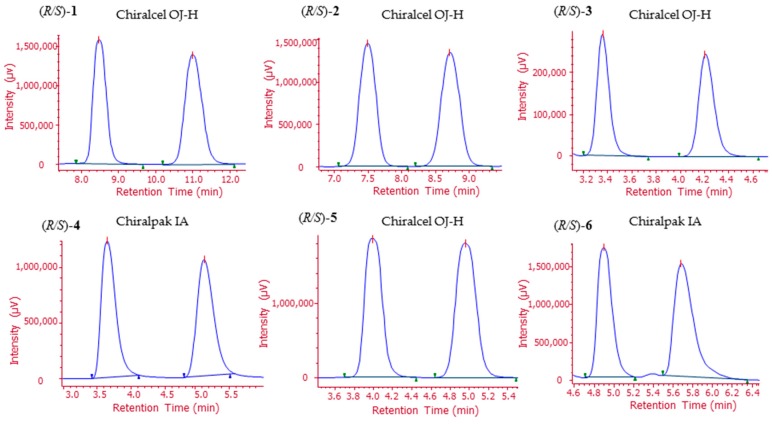
Analytical enantiomer separation of (*R*/*S*)-**1**, (*R*/*S*)-**2**, (*R*/*S*)-**3**, (*R*/*S*)-**5** on Chiralcel OJ-H (4.6 mm × 150 mm, dp = 5 μm) and of (*R*/*S*)-**4** and (*R*/*S*)-**6** on Chiralpak IA (4.6 mm × 250 mm, dp = 5 μm). (*R*/*S*)-**1** elution condition: 100% EtOH, DEA 0.1%, flow rate 0.5 mL/min. (*R*/*S*)-**2**, (*R*/*S*)-**3** and (*R*/*S*)-**5** elution condition: 100% MeOH, DEA 0.1%, flow rate 1.0 mL/min. (*R*/*S*)-**4** and (*R*/*S*)-**6** elution condition: 100% MeOH, 0.1% DEA, flow rate 1 mL/min. For all: injection volume 10 μL.

To sum up, we set up methods able to give baseline separation of **1**–**6** in a relatively short analysis time. In detail, the methods foresee the use of Chiralpak IA for solving Compounds **4** and **6** and of Chiralcel OJ-H for solving **1**–**3** and **5**. In view of these results, we considered the developed methodologies suitable for the scale-up to the (semi-)preparative scale, and accordingly, no further attempts were made to extend the screening under HPLC conditions. Figure 2 shows chromatograms of racemic **1**–**6**, when the highest resolution was reached, that is using Chiralpak IA or Chiralcel OJ-H columns, eluting with 100% methanol (Compounds **2**–**6**) or with 100% ethanol (Compound **1**).

### 2.3. Preparation of Enantiomeric ***1***–***6*** through HPLC

During the drug discovery process, (semi-)preparative resolution of enantiomers using HPLC is a powerful technique for the rapid preparation of enantiomers. Employing this technique, important prerequisites for an economic and productive preparative enantiomer separation are: (i) retention times as short as possible; (ii) high solubility of the racemate and the enantiomers in the eluent/injection solvent; and (iii) the use of a mobile phase consisting of a pure low-cost solvent, facilitating work-up and re-use of the mobile phase. As previously discussed, using Chiralpak IA or Chiralcel OJ-H columns and eluting with alcohols added with DEA (0.1%), relatively short retention times (less than 12 min for the second eluted enantiomer), high enantioselectivity and good resolution could be observed (Figure 2). Accordingly, these experimental conditions were transferred to a Chiralpak IA and a Chiralcel OJ-H columns with an ID of 10 mm, on which a maximum of 12.5 mg could be separated in one run, depending on the solubility of the compound in the mobile phase. Therefore, racemic **1**–**6** were processed in a low number of cycles (Table 6), leading to enantiopure **1**–**6** in satisfactory amounts and yields, with an ee ≥ 95% (Table 6), as evidenced by analytical control of the collected fractions (Figure S1).

**Figure 3:**
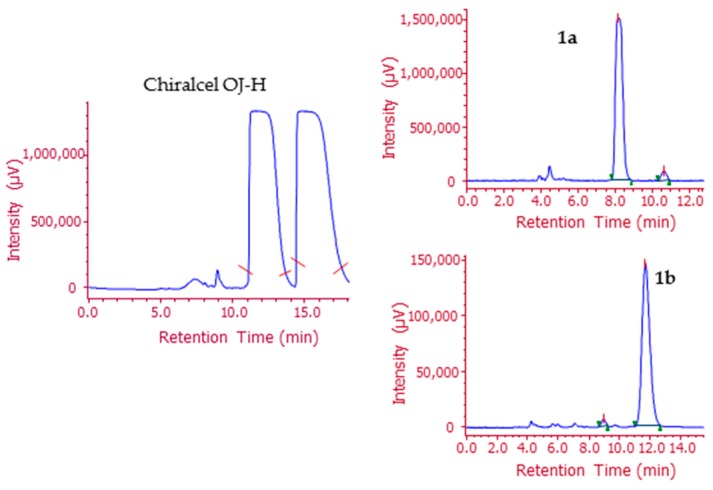
Representative chromatogram of (semi-)preparative resolution: compound (*R*/*S*)-**1,** Chiralcel OJ-H (10 mm × 250 mm, dp = 5 μm). Elution condition: 100% EtOH, DEA 0.1%, flow rate 2.5 mL/min. Injection volume 1.0 mL. **1a**: (+)-**1**, **1b**: (−)-**1**.

A representative example of (semi-)preparative resolution is reported in Figure 3. Actually, 30 mg of racemic **1** were processed in three runs (45 min in total) at a flow rate of 2.5 mL/min at room temperature. According to the chromatographic profile (Figure 3), a small intermediate fraction was collected. Final analysis of **1** enantiomers is shown in Figure 3.

### 2.4. Absolute Configurational Assignment

As the last step of this work, the absolute configuration at the stereogenic center of enantiomeric **1**–**6** was established by combining electronic circular dichroism (ECD) and chiral HPLC analysis. The electronic circular dichroism (ECD) spectra have been compared with the ECD curves of structural analogues RC-33 and RC-34, whose configuration was already assigned by us [13,14]. In detail, the ECD spectra of enantiomeric arylamino alcohols **1**, **3** and **5** were compared with that of (*S*)-RC-34 (Figure 1) and the ECD spectra of enantiomeric **2**, **4** and **6**, with that of (*S*)-RC-33.

**Figure 4:**
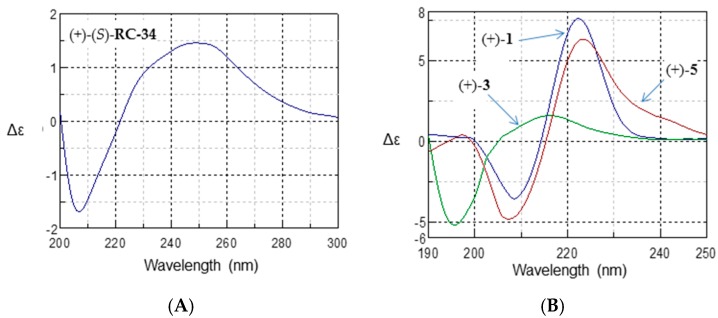
ECD curves of (**A**) (+)-(*S*)-RC-34 and (**B**) (+)-**1**, (+)-**3**, (+)-**5** and their Cotton effect (CE) in the wavelength range 190–300 nm.

Briefly, comparable Cotton effects (CEs) for (+)-**1**, (+)-**3**, (+)-**5** and (+)-(*S*)-RC-34 compounds are evident in two ranges of wavelengths. Between 195 and 207 nm, there are negative CEs, and between 216 and 223 nm there are positive CE, attributable to ^1^*L*_a_ and ^1^*L*_b_ electronic transitions of benzene and naphthalene (Figure 4). The sign of the CEs of ^1^*L*_a_ is consistently opposite that at the longer wavelength (^1^*L*_b_). Some evident differences in the profile of the curves are in agreement with the UV spectra and could depend on the substitution pattern of the aromatic moiety. Basing on these considerations, the (*S*) absolute configuration of (+)-(*S*)-RC-34 may be proposed also for (+)-**1**, (+)-**3** and (+)-**5**, because the sequence around the stereogenic center is the same for all four compounds.

Further support to this configurational assignment was derived from the study of the elution order of chiral HPLC analysis, taking into account that the absolute configuration of structurally-related compounds usually follows the same chiral recognition mechanism in the chromatographic process on a given chiral stationary phase, using the same mobile phase. Accordingly, (*S*)-RC-34, (+)-**1**, (+)-**3** and (+)-**5** were analyzed under the same chromatographic conditions (Chiralcel OJ-H, 50% MeOH and 50% EtOH, 0.1% DEA) and resulted in all cases in the first eluted enantiomers. The elution order results confirmed that the (*S*) absolute configuration may be attributed also to (+)-**1**, (+)-**3** and (+)-**5**.

**Figure 5:**
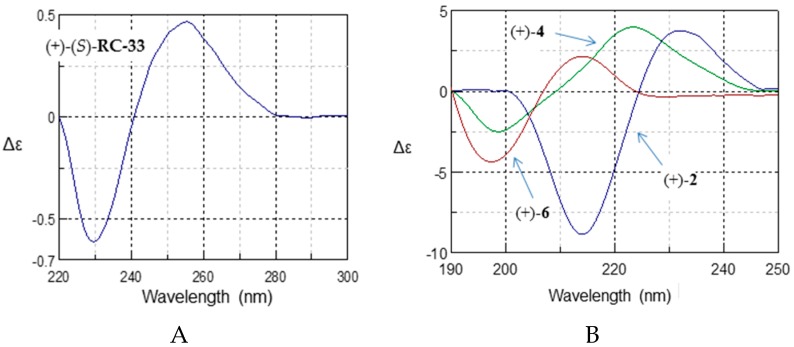
ECD curves of (**A**) (+)-(*S*)-RC-33, **B**) (+)-**2**, (+)-**4**, (+)-**6** and their Cotton effect (CE) in the wavelength range of 190–300 nm.

Empirical absolute configuration assignment of **2**, **4**, **6** was effected applying a similar approach. ECD spectra of **2**, **4**, **6** have been compared with that of the structurally-related compound (*S*)-RC-33, as a reference of known stereochemistry. The enantiomers (+)-**2**, (+)-**4**, (+)-**6** and (*S*)-RC-33 displayed a similar negative CE in the wavelength range between 198 and 230 nm and a CE between 214 and 255 nm, associated with ^1^*L*_a_ and ^1^*L*_b_ electronic transitions of the aromatic chromophores, respectively. Again, the sign of the CEs of ^1^*L*_a_ is consistently opposite that at the longer wavelength (^1^*L*_b_), and the differences in the profile of the curves could depend on the different aromatic nucleus. Based on these considerations, the (*S*) absolute configuration of (+)-(*S*)-RC-33 may be proposed also for (+)-**2**, (+)-**4** and (+)-**6** (Figure 5), having the same substituents around the stereogenic center. Again, (*S*)-RC-33 and (+)-**2** were analyzed under the same chromatographic conditions (Chiralcel OJ-H, 50% MeOH and 50% EtOH, 0.1% DEA) and resulted in both cases in the first eluted enantiomers. Unfortunately, the OJ-H column is not able to solve Compounds **4** and **6**. To confirm that a set of structurally-related compounds is characterized by the same absolute configuration, they must be analyzed under the same chromatographic conditions. Indeed, chiral recognition mechanisms on chiral stationary phases may be sensitive to even minor structural or conditional changes. Therefore, we took into consideration the chromatographic profiles of (*S*)-RC-33, (+)-**2**, (+)-**4** and (+)-**6** on Chiralpak IC (90/10 *n*-hep/IPA, 0.1% DEA), a condition that ensures the baseline separation of all of the studied compound enantiomers. (*S*)-RC-33, (+)-**2**, (+)-**4** and (+)-**6** showed the same behaviors, confirming that all of the first eluted enantiomers are characterized by the same absolute configuration, that is the (*S*) configuration. These results are in agreement with the data collected through ECD technique, and the (*S*) absolute configuration may be attributed also to (+)-**1**, (+)-**3** and (+)-**5**.

## 3. Experimental Section

### 3.1. HPLC-UV Chiral Resolution

In order to identify the best conditions to be properly scaled up to the (semi-)preparative scale, an analytical screening of cellulose- and amylose-based CSPs was firstly performed using Chiralcel OJ-H (Chiral technologies Europe, Illkirch-Cedex, France, Europe, 4.6 mm diameter × 150 mm length, dp 5 μm), Chiralpack IC (Chiral technologies Europe, Illkirch-Cedex, France, Europe, 4.6 mm diameter × 250 mm length, dp 5 μm) and Chiralpack IA (Chiral technologies Europe, Illkirch-Cedex, France, Europe, 4.6 mm diameter × 250 mm length, dp 5 μm) columns and eluting with a flow rate of 0.5 mL/min, 1 mL/min and 1 mL/min, respectively. Analytes were detected photometrically at 220 and 254 nm. Unless otherwise specified, sample solutions were prepared dissolving analytes at 1 mg/mL in ethanol and filtered through 0.45-μm PTFE membranes before analysis. The injection volume was 10 μL. The mobile phases consisted of alcohols (MeOH or/and EtOH) or mixtures of *n*-hep and polar modifiers (EtOH or IPA). In all cases, 0.1% of DEA was added to the mobile phase. In the case of the Chiralpak IC columns, the analyses were carried out also in the presence of 0.3% of TFA. The retention factor (*k*) was calculated using the equation *k* = (t_R_ − t_0_)/t_0_, where t_R_ is the retention time and t_0_ the dead time (t_0_ was considered to be equal to the peak of the solvent front and was taken from each particular run). The enantioselectivity (α) and the resolution factor (Rs) were calculated as follows: α = *k*_2_/*k*_1_ and Rs = 2 (t_R2_ − t_R1_)/(w_1_ + w_2_), where t_R2_ and t_R1_ are the retention times of the second and the first eluted enantiomers and w_1_ and w_2_ are the corresponding base peak widths. The best conditions found by the screening protocol were applied to a (semi-)preparative scale-up. The enantiomers of **1**, **2**, **3** and **5** were then completely resolved by a (semi-)preparative process using a Chiralcel OJ-H column (10 mm diameter × 250 mm length, 5 μm), eluting with EtOH (for Compound **1**) or MeOH (for Compounds **2**, **3** and **5**) at rt with a flow rate of 2.5 mL/min. Compounds **4** and **6** were resolved on Chiralpak IA (10 mm diameter × 250 mm length, 5 μm) using MeOH at a flow rate of 2.5 mL/min as the eluent.

The eluate was fractioned according to the UV profile (detection at 220 and 254 nm). The fractions obtained containing the enantiomers were evaporated at reduced pressure. Analytical control of the collected fractions was performed using the analytical columns.

### 3.2. Electronic Circular Dichroism

The solutions of **1**–**6** enantiomers: (+)-**1** (*c*: 2.68 × 10^−5^ M, in *n*-hexane), (+)-**2** (*c*: 1.90 × 10^−5^ M in *n*-hexane), (+)-3 (*c*: 3.09 × 10^−5^ M in *n*-hexane), (+)-4 (*c*: 2.21 × 10^−5^ M in *n*-hexane), (+)-5 (*c*: 6.29 × 10^−6^ M in *n*-hexane), (+)-6 (*c*: 1.31 × 10^−5^ M in *n*-hexane; optical pathway 1 cm), (+)-(*S*)-RC-34 (*c*: 2.5 × 10^−5^ M in *n*-hexane; optical pathway 1 cm) and (+)-(*S*)-RC-33 (*c*: 9.12 × 10^−5^ M in *n*-hexane; optical pathway 1 cm) were analyzed in a nitrogen atmosphere. ECD spectra were scanned at 50 nm/min with a spectral band width of 2 nm and a data resolution of 0.5 nm (Figure 4 and Figure 5).

## 4. Conclusions

In this paper, we presented the synthesis of the novel potential SR modulators **1**–**6** and their enantioseparation via HPLC chiral resolutions on cellulose- and amylose-based CSPs. A systematic screening protocol for enantioselective HPLC was established leading to fast and easy-to-use chiral HPLC separations suitable for a (semi-)preparative scale-up. The separation of the enantiomers was optimized by varying the chromatographic parameters. Baseline separations were obtained for all of the studied compounds under optimized chromatographic conditions. The recovery of the enantiomers after chromatography was in the range of 76%–95%, for the individual enantiomers. (Semi-)preparative enantioselective chromatography for compounds of interest proves to be a straightforward, productive and robust methodology for the quick access to the desired amounts of pure enantiomers. It remains one of the most versatile and cost effective tools for the fast isolation of desired enantiomers from a racemic mixture. The absolute configuration at the chiral center of enantiomeric **1**–**6** was empirically assigned by combining electronic circular dichroism (ECD) and chiral HPLC analysis.

The bioactivity of each enantiomer is now under investigation in order to deeply understand the role of chirality in the SRs-ligand interaction.

## Figures and Tables

**Table:** 

Compound	λ_min_	Δε_min_	λ_max_	Δε_max_
(*S*)-RC-34	206.5	−2.78	253.0	1.09
(+)-**1**	208.5	−3.58	222.5	7.59
(+)-**3**	195.5	−5.19	216.5	1.57
(+)-**5**	207.5	−4.82	223.5	6.31

**Table:** 

Compound	λ_min_	Δε_min_	λ_max_	Δε_max_
(*S*)-RC-33	229.8	−0.61	255.0	0.47
(+)-**2**	214.0	−9.04	232.5	3.56
(+)-**4**	198.5	−2.26	223.0	3.85
(+)-**6**	198.0	−4.34	214.0	2.18

**Table 1:**
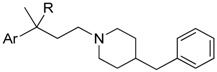
Molecules under investigation.

Compound	Ar	R
**1**	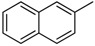	OH
**2**		H
**3**	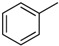	OH
**4**		H
**5**	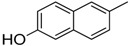	OH
**6**		H

**Table 2:** Screening protocol: mobile phase composition.

Entry	Mobile Phase Composition (%)			
	MeOH	EtOH	*n-*hep	IPA
1	100	-	-	-
2	50	50	-	-
3	-	10	90	-
4	-	-	90	10

**Table 3:** Analytical screening on Chiralpak IC with different mobile phases containing 0.1% DEA and 0.3% TFA.

Compound	Mobile Phase			K_1_	K_2_	Α	Rs
	*n*-hep (%)	EtOH (%)	IPA (%)				
(*R*/*S*)-**1**	90	10	-	1.52		1	-
	90	-	10	4.45		1	-
	92	-	8	7.76	8.72	1.12	1.44
(*R*/*S*)-**2**	90	10	-	1.93		1	-
	90	-	10	7.83	9.62	1.23	2.36
(*R*/*S*)-**3**	90	10	-	1.29		1	-
	90	-	10	1.35		1	-
(*R*/*S*)-**4**	90	10	-	1.56		1	-
	90	-	10	6.52	8.68	1.33	3.08
(*R*/*S*)-**5**	90	10	-	3.24		1	-
	90	-	10	15.79		1	-
(*R*/*S*)-**6**	90	10	-	4.07		1	-
	90	-	10	27.14	29.97	1.11	1.26

Flow rate: 1 mL/min. Detection: 254 nm (Compounds **1**, **2**, **5**, **6**) and 220 nm (Compounds **3**, **4**).

**Table 4:** Analytical screening on Chiralpak IA with different mobile phases containing 0.1% DEA.

Compound	Mobile Phase				K_1_	K_2_	α	Rs
	*n*-hep (%)	MeOH (%)	EtOH (%)	IPA (%)				
(*R*/*S*)-**1**	-	100	-	-	5.48		1	-
	90	-	10	-	3.48		1	-
	90	-	-	10	5.00	5.55	1.11	1.69
(*R*/*S*)-**2**	-	100	-	-	1.00	1.24	1.24	1.73
	90	-	10	-	2.62		1	-
	90	-	-	10	4.24	5.07	1.19	2.08
(*R*/*S*)-**3**	-	100	-	-	0.53		1	-
	90	-	10	-	2.77	3.25	1.17	1.67
	90	-	-	10	3.84		1	-
(*R*/*S*)-**4**	-	100	-	-	0.57	0.81	1.42	3.06
	90	-	10	-	1.99		1	-
	90	-	-	10	3.21		1	-
(*R*/*S*)-**5**	-	100	-	-	0.35		1	-
	90	-	10	-	13.72	17.38	1.27	3.04
	90	-	-	10	14.68	18.55	1.26	2.95
(*R*/*S*)-**6**	-	100	-	-	0.96	1.27	1.32	2.49
	90	-	10	-	8.90	10.45	1.18	3.22
	90	-	-	10	19.76	23.86	1.21	2.94

Flow rate: 1 mL/min. Detection: 254 nm (Compounds **1**, **2**, **5**, **6**) and 220 nm (Compounds **3**, **4**).

**Table 5:** Analytical screening on Chiralcel OJ-H with different mobile phases containing 0.1% DEA.

Compound	Mobile Phase				*K*_1_	*K*_2_	α	Rs
	*n*-hep (%)	MeOH (%)	EtOH (%)	IPA (%)				
(*R*/*S*)-**1**	-	100	-	-	2.5		1	-
	-	50	50	-	1.77	2.40	1.36	3.14
	-	-	100	-	1.03	1.63	1.58	3.25
	90	-	-	10	1.56	2.27	1.46	2.35
(*R*/*S*)-**2**	-	100	-	-	3.98	4.80	1.21	2.46
	-	50	50	-	1.87	2.25	1.20	1.97
	-	-	100	-	1.23	1.46	1.19	1.83
	90	-	-	10	9.61		1	-
(*R*/*S*)-**3**	-	100	-	-	1.24	1.80	1.45	4.05
	-	50	50	-	0.70	0.93	1.33	2.32
	-	-	100	-	2.24		1	-
	90	-	-	10	0.46		1	-
(*R*/*S*)-**4**	-	100	-	-	1.52		1	-
	-	50	50	-	0.74		1	-
	-	-	100	-	0.46		1	-
	90	-	-	10	0.54		1	-
(*R*/*S*)-**5**	-	100	-	-	1.10	1.61	1.46	2.62
	-	50	50	-	0.74	1.07	1.45	1.99
	-	-	100	-	0.39	0.70	1.79	1.92
	90	-	-	10	0.32		1	-
(*R*/*S*)-**6**	-	100	-	-	2.20		1	-
	-	50	50	-	1.25		1	-
	-	-	100	-	0.73		1	-
	90	-	-	10	0.61		1	-

Flow rate: 0.5 mL/min. Detection: 254 nm (Compounds **1**, **2**, **5**, **6**) and 220 nm (Compounds **3**, **4**).

**Table 6:** (Semi-)preparative resolution of (*R*/*S*)-**1**, (*R*/*S*)-**2**, (*R*/*S*)-**3**, (*R*/*S*)-**5** on Chiralcel OJ-H (10 mm × 250 mm, dp = 5 μm) and of (*R*/*S*)-**4** and (*R*/*S*)-**6** on Chiralpak IA (10 mm × 250 mm, dp = 5 μm).

Compound	CSP	Processed Amount (mg)	No. Cycles	Enantiomer	Isolated Amount (mg)	ee (%)	Yield (%)	[α]${}_{D}^{20}$ (MeOH)
(*R*/*S*)-**1**	Chiralcel OJ-H	30	3	(+)-**1**	14.1	96.0	94.0	+40.5
				(−)-**1**	14.3	97.0	95.3	−42.3
(*R*/*S*)-**2**	Chiralcel OJ-H	30	3	(+)-**2**	13.8	95.0	92.0	+6.1
				(−)-**2**	12.5	95.0	83.3	−6.3
(*R*/*S*)-**3**	Chiralcel OJ-H	50	4	(+)-**3**	22.9	99.9	91.6	+10.5
				(−)-**3**	23.0	98.0	92.0	−9.2
(*R*/*S*)-**4**	Chiralpak IA	16	2	(+)-**4**	6.1	99.9	76.3	+8.2
				(−)-**4**	6.3	99.9	78.8	−8.3
(*R*/*S*)-**5**	Chiralcel OJ-H	22	4	(+)-**5**	9.1	99.9	82.7	+24.2
				(−)-**5**	8.9	99.9	80.9	−24.8
(*R*/*S*)-**6**	Chiralpak IA	25	3	(+)-**6**	10.5	99.9	84.0	+11.8
				(−)-**6**	9.8	99.9	78.4	−12.0

Flow rate: 2.5 mL/min. Detection: 254 nm (Compounds **1**, **2**, **5**, **6**) and 220 nm (Compounds **3**, **4**). Injection volume: 1.0 mL.

## Supplementary Materials

Supplementary materials can be accessed at: http://www.mdpi.com/1420-3049/21/9/1210/s1.

## Abbreviations

The following abbreviations are used in this manuscript:

MDPI	Multidisciplinary Digital Publishing Institute
DOAJ	Directory of Open Access Journals
DEA	Diethylamine
ECD	Electronic circular dichroism
EtOH	Ethanol
IPA	Isopropanol
MeOH	Methanol
*n*-hep	*n*-Heptane
TFA	Trifluoroacetic acid
UV	Ultraviolet
