# Supplementary material for: Novel Enantiopure Sigma Receptor Modulators: Quick (Semi-)Preparative Chiral Resolution via HPLC and Absolute Configuration Assignment
Source: Molecules. 2016 Sep 10;21(9):1210. doi: 10.3390/molecules21091210 (PMC6273946; doi:10.3390/molecules21091210)
Supplement: Supplementary file 1 [file molecules-21-01210-s001.pdf]

Supplementary Materials: Novel Enantiopure Sigma Receptor (SR) Modulators: Quick (Semi)-Preparative Chiral Resolution via HPLC and Absolute Configuration Assignment

Marta Rui, Annamaria Marra, Vittorio Pace, Markus Juza, Daniela Rossi and Simona Collina

1. Materials and Methods

1.1. General

Optical rotation values were measured on a Jasco photoelectric polarimeter DIP 1000 using a 0.5-dm cell and a sodium and mercury lamp (λ = 589 nm, 435 nm, 405 nm); sample concentration values (c) are given in 10^{-2} g·mL^{-1}.

Proton nuclear magnetic resonance (NMR) spectra were recorded on a Bruker Avance 500 spectrometer operating at 500 MHz. Proton chemical shifts (δ) are reported in ppm with the solvent reference relative to tetramethylsilane (TMS) employed as the internal standard (CDCl3, δ = 7.26 ppm). The following abbreviations are used to describe spin multiplicity: s = singlet, d = doublet, t = triplet, q = quartet, m = multiplet, br = broad signal, dd = doublet-doublet, td = triplet-doublet. The coupling constant values are reported in Hz. ^{13}C-NMR spectra were recorded on a 500-MHz spectrometer, with complete proton decoupling. Carbon chemical shifts (δ) are reported in ppm relative to TMS with the respective solvent resonance as the internal standard (CDCl3, δ = 77.23 ppm). UPLC-UV-ESI/MS analyses were carried out on an Acquity UPLC Waters LCQ FLEET system using an ESI source operating in positive ion mode, controlled by ACQUITY PDA and 4 MICRO (Waters S.A.S., Saint-Quentin En Yvelines Cedex, France). Analyses were run on a ACQUITY BEH C18 (Waters S.A.S). 50 × 2.1 mm, 1.7 μm) column, at room temperature, with gradient elution (Solvent A: water containing 0.1% of formic acid; Solvent B: methanol containing 0.1% of formic acid; gradient: 10% B in A to 100% B in 3 min, followed by isocratic elution 100% B for 1.5 min, return to the initial conditions in 0.2 min) at a flow rate of 0.5 mL·min^{-1}. All of the final compounds had 95% or greater purity.

1.2. Compound Characterization

(+)-(*S*)-4-(4-Benzyl-piperidin-1-yl)-2-naphthalen-2-yl-butan-2-ol, [(+)-(*S*)-1]: White solid; $[\alpha]_D^{20}$ = +40.5 (c 0.2, CH_3OH). IR (cm^{-1}): 3434, 2918, 1653, 1438, 1156, 1112, 820; ^{1}H-NMR (500 MHz) ($CDCl_3$) δ (ppm): 8.01 (s, 1H), 7.86–7.81 (m, 3H), 7.47–7.45 (m, 3H), 7.28 (t, *J* = 7.8 Hz, 2H), 7.19 (t, *J* = 7.0 Hz, 1H), 7.13 (d, *J* = 7.1 Hz, 2H), 3.19 (d, 1H), 2.54–2.50 (m, 3H), 2.31 (m, 1H), 2.22–2.14 (m, 2H), 1.92 (d, 1H), 1.84 (m, 1H), 1.76 (m, 1H), 1.68 (m, 1H), 1.60 (m, 1H), 1.57 (s, 3H), 1.50 (m, 1H), 1.31 (m, 2H); ^{13}C-NMR (500 MHz) ($CDCl_3$) δ (ppm): 146.3, 140.5,133.3, 132.0, 129.1, 128.2, 128.1, 127.6, 127.4, 125.8, 125.4, 123.8, 123.6, 75.7, 55.1, 54.8, 52.6, 43.1, 37.8, 37.4, 32.6, 32.1, 31.4. HPLC: t_R = 8.5 min, ee 96.0%.

(−)-(*R*)-4-(4-Benzyl-piperidin-1-yl)-2-naphthalen-2-yl-butan-2-ol, [(−)-(*R*)-1]: White solid; $[\alpha]_D^{20}$ = −42.3 (c 0.2, CH_3OH). The IR and NMR spectra are identical to that of (+)-(*S*)-**1**. HPLC: t_R = 11.1 min, ee 97.0%.

(+)-(*S*)-4-Benzyl-1-(3-naphthalen-2-yl-butyl)-piperidine, [(+)-(*S*)-2]: Yellow oil; $[\alpha]_D^{20}$ = +6.1 (c 0.2, CH_3OH). IR (cm^{-1}): 3025, 2924, 2508, 1631, 1602, 1542, 1496, 1453; ^{1}H-NMR (500 MHz) ($CDCl_3$) δ (ppm): 7.79–7.77 (t, *J* = 8.7 Hz, 3H), 7.59 (s, 1H), 7.45-7.42 (m, 2H), 7.32 (d, *J* = 8.8 Hz, 1H), 7.25 (m, 2H), 7.17 (m, 1H), 7.08 (m, 2H), 3.30 (broad peak, 2H), 2.90 (m, 1H), 2.76 (m, 1H), 2.55 (d, *J* = 7.4 Hz, 2H), 2.46 (m, 1H), 2.27–2.13 (m, 4H), 1.82–1.71 (m, 4H), 1.59 (m, 1H), 1.36 (d, *J* = 6.5 Hz, 3H); ^{13}C-NMR (500 MHz) ($CDCl_3$) δ (ppm): 142.4, 139.3, 133.4, 132.3, 128.9, 128.5, 128.3, 127.6, 127.5, 126.1, 125.5, 125.3, 124.8, 56.1, 52.4, 41.9, 38.3, 36.5, 31.8, 29.1, 22.7. HPLC: t_R = 7.7 min, ee 95.0%.

(−)-(*R*)-4-Benzyl-1-(3-naphthalen-2-yl-butyl)-piperidine, [(−)-(*R*)-2]: Yellow oil; $[\alpha]_D^{20}$ = −6.3 (c 0.2, CH_3OH). The IR and NMR spectra are identical to that of (+)-(*S*)-**2**. HPLC: t_R = 9.0 min, ee 95.0%.

(+)-(*S*)-4-(4-Benzyl-piperidin-1-yl)-2-phenyl-butan-2-ol, [(+)-(*S*)-3]: Yellow oil; $[\alpha]_D^{20}$ = +10.5 (c 0.6, CH_3OH). IR (cm^{-1}): 3184, 3125, 1602, 1369, 1343, 1156, 846, 699; ^{1}H-NMR (500 MHz) ($CDCl_3$) δ (ppm): 7.45 (d, *J* = 8.9 Hz, 2H), 7.33 (t, *J* = 8.1 Hz, 2H), 7.27 (t, *J* = 7.4 Hz, 2H), 7.21-7.19 (m, 2H), 7.13 (d, *J* = 7.0 Hz, 2H), 3.15 (d, 1H), 2.54 (m, 1H), 2.52 (m, 2H) 2.30 (m, 1H), 2.22 (m, 1H), 2.07 (m, 1H), 1.86 (m, 1H), 1.80 (m, 1H) 1.75 (m, 1H), 1.67 (m, 1H) 1.60 (m, 1H), 1.50 (m, 1H), 1.49 (s, 3H) 1.31 (m, 2H); ^{13}C-NMR (500 MHz) ($CDCl_3$) δ (ppm): 148.9, 140.5, 129.1, 127.9, 128.2, 126.0, 125.8, 125.0, 75.5, 55.1, 54.8, 52.6, 43.1, 37.8, 37.7, 32.6, 32.1, 31.4. HPLC: t_R = 3.4 min, ee 99.9%.

(−)-(*R*)-4-(4-Benzyl-piperidin-1-yl)-2-phenyl-butan-2-ol, [(−)-(*R*)-3]: Yellow oil; $[\alpha]_D^{20}$ = −9.2 (c 0.6, CH_3OH). The IR and NMR spectra are identical to that of (+)-(*S*)-**3**. HPLC: t_R = 4.2 min, ee 98.0%.

(+)-(*S*)-4-Benzyl-1-(3-phenyl-butyl)-piperidine, [(+)-(*S*)-4]: Yellow oil; $[\alpha]_D^{20}$ = +8.2 (c 0.3, CH_3OH). IR (cm^{-1}): 3682, 3019, 2929, 2856, 2434, 2400, 1230; ^{1}H-NMR (500 MHz) ($CDCl_3$) δ (ppm): 7.28–7.27 (m, 4H), 7.17 (m, 4H), 7.13 (d, *J* = 7.0 Hz, 2H), 2.86 (broad peak, 2H), 2.71 (m, 1H), 2.52 (d, *J* = 6.6 Hz, 2H), 2.26 (m, 1H), 2.14 (m, 1H), 1.87–1.72 (m, 4H), 1.61 (d, 2H), 1.49 (m, 1H), 1.29 (m, 2H), 1.24 (d, *J* = 7.3 Hz, 3H); ^{13}C-NMR (500 MHz) ($CDCl_3$) δ (ppm): 147.3, 140.7, 129.1, 128.3, 128.1, 126.9, 12 5.9, 125.7, 57.3, 54.1, 53.9, 43.2, 38.4, 37.9, 35.4, 32.1, 22.6. HPLC: t_R = 3.7 min, ee 99.9%.

(−)-(*R*)-4-Benzyl-1-(3-phenyl-butyl)-piperidine, [(−)-(*R*)-4]: Yellow oil; $[\alpha]_D^{20}$ = −8.3 (c 0.3, CH_3OH). The IR and NMR spectra are identical to that of (+)-(*S*)-**4**. HPLC: t_R = 5.3 min, ee 99.9%.

(+)-(*S*)-6-[3-(4-Benzyl-piperidin-1-yl)-1-hydroxy-1-methyl-propyl]-naphthalen-2-ol, [(+)-(*S*)-5]: Yellow oil; $[\alpha]_D^{20}$ = +24.2 (c 0.1, CH_3OH). IR (cm^{-1}): 3452, 2925, 1633, 1605, 1560, 1454, 1381; ^{1}H-NMR (500 MHz) ($CDCl_3$) δ (ppm): 7.91 (s, 1H), 7.72 (d, *J* = 8.4 Hz, 1H), 7.60 (d, *J* = 8.4 Hz, 1H), 7.40 (d, *J* = 8.8 Hz, 1H), 7.25 (t, *J* = 6.9 Hz, 2H), 7.18–7.14 (m, 3H), 7.07 (d, *J* = 8.0 Hz, 2H), 3.20 (broad peak, 1H), 2.61 (broad peak, 1H), 2.46 (d, *J* = 6.5 Hz, 2H), 2.34 (m, 2H), 2.18 (m, 1H), 1.96 (broad peak, 1H), 1.88 (broad peak, 1H), 1.80 (broad peak, 1H), 1.66 (broad peak, 2H), 1.59 (s, 1H), 1.49 (broad peak, 1H), 1.34 (broad peak, 2H); ^{13}C-NMR (500 MHz) ($CDCl_3$) δ (ppm): 154.0, 143.0, 140.3, 133.3, 129.8, 129.0, 128.5, 128.2, 126.2, 125.8, 124.1, 123.4, 118.3, 109.2, 75.8, 55.0, 54.7, 52.6, 42.9, 37.6, 37.4, 31.7, 31.3. HPLC: t_R = 4.0 min, ee 99.9%.

(−)-(*R*)-6-[3-(4-Benzyl-piperidin-1-yl)-1-hydroxy-1-methyl-propyl]-naphthalen-2-ol, [(−)-(*R*)-5]: Yellow oil; $[\alpha]_D^{20}$ = −24.8 (c 0.1, CH_3OH). The IR and NMR spectra are identical to that of (+)-(*S*)-**5**. HPLC: t_R = 5.0 min, ee 99.9%.

(+)-(*S*)-6-[3-(4-Benzyl-piperidin-1-yl)-1-methyl-propyl]-naphthalen-2-ol, [(+)-(*S*)-6]: Yellow oil; $[\alpha]_D^{20}$ = +11.8 (c 0.3, CH_3OH). IR (cm^{-1}): 3297, 2924, 2349, 2309, 1604, 1453, 1376, 1269; ^{1}H-NMR (500 MHz) ($CDCl_3$) δ (ppm): 7.42 (d, *J* = 9.0 Hz, 1H), 7.37 (s, 1H), 7.24 (t, 2H), 7.17 (d, *J* = 8.0 Hz, 1H), 7.17 (m, 1H), 7.06 (m, 3H), 6.98 (d, *J* = 9.0 Hz, 1H), 6.81 (s, 1H), 3.26 (broad peak, 1H), 3.10 (broad peak, 1H), 2.73 (m, 1H), 2.50–2.49 (broad peak, 4H), 2.14–2.12 (m, 2H), 2.04 (m, 2H), 1.67 (m, 2H), 1.60–1.54 (m, 3H), 1.28 (overlapped peak, 3H); ^{13}C-NMR (500 MHz) ($CDCl_3$) δ (ppm): 154.6, 139.5, 133.6, 129.0, 128.3, 126.8, 126.0, 125.1, 125.0, 118.7, 109.2, 56.5, 53.7, 52.9, 42.3, 38.1, 37.0, 33.2, 30.0, 22.9. HPLC: t_R = 4.9 min, ee 99.9%.

(−)-(*R*)-6-[3-(4-Benzyl-piperidin-1-yl)-1-methyl-propyl]-naphthalen-2-ol, [(−)-(*R*)-6]: Yellow oil; $[\alpha]_D^{20}$ = −12.0 (c 0.3, CH_3OH). The IR and NMR spectra are identical to that of (+)-(*S*)-**6**. HPLC: t_R = 5.7 min, ee 99.9%.

1.3. (Semi-)Preparative Chromatograms and Analytical Controls

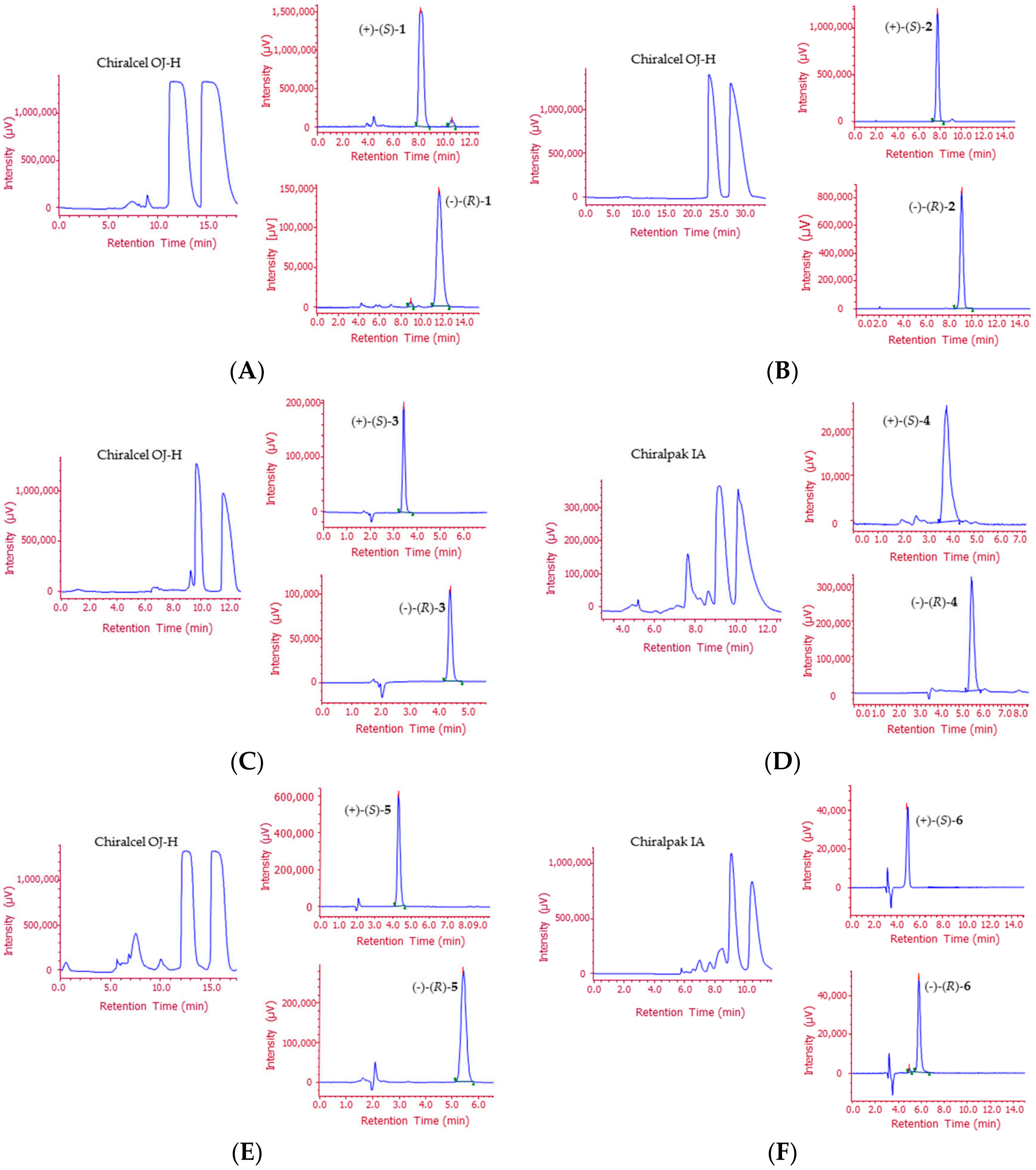

Figure S1. (Semi-)preparative enantiomer separations and analytical enantioselective analysis of first and second collected fractions of (**A**) **1**, (**B**) **2**, (**C**) **3**, (**D**) **4**, (**E**) **5**, (**F**) **6**. Elution conditions: (**A**) 100% EtOH, DEA 0.1%, flow rate 2.5 mL/min for (semi-)preparative analysis and 0.5 mL/min for analytical analysis; (**B**, **C**, **E**) 100% MeOH, DEA 0.1%, flow rate 2.5 mL/min for (semi-)preparative analysis and 0.5 mL/min for analytical analysis; and (**D**, **F**) 100% MeOH, 0.1% DEA, flow rate 2.5 mL/min for (semi-)preparative analysis and 1 mL/min for analytical analysis. Injection volume: 1 mL for (semi-)preparative analysis and 10 µL for analytical analysis. Detection: 254 nm (Compounds **1**, **2**, **5**, **6**) and 220 nm (Compounds **3**, **4**).
